# Research on layout model and construction planning of aged care institutions for disabled elders in China: based on Nanjing city data

**DOI:** 10.1186/s12877-023-03924-z

**Published:** 2023-04-20

**Authors:** Xiaojun Guo, Houxue Shen, Qinglan Wen, Sifeng Liu, Yingjie Yang, Hui Zhang

**Affiliations:** 1 School of Science, Nantong University, 9 Seyuan Road, Nantong, 226019 China; 2 College of Economics and Management, Nanjing University of Aeronautics and Astronautics, Nanjing, 211106 China; 3 Institute of Artificial Intelligence, De Montfort University, Leicester, LE1 9BH UK

**Keywords:** Active aging, Layout of aged care institutions, Construction planning, Multi-objective programming, Genetic algorithm, Multiple attribute decision making

## Abstract

**Background:**

To meet the needs of diversified pension services and the construction of aged care institutions is one of the urgent livelihood issues in China. Under the major national needs of pension and care for the elderly, it is necessary to optimize the allocation of pension and care for the elderly resources, and formulate operational coping strategies and optimization plans. It is of great significance to deal with the urgent problem of population aging in a timely and scientific way.

**Methods:**

The economic benefits and social costs of aged care institutions are regarded as objective functions. To satisfy the economic benefits, it is necessary to reduce the social costs, and its construction quantity can only be an integer. The multi-objective genetic algorithm is improved with integer programming, and the global optimal solution is achieved after several times of searching. Under the multi-objective optimization model, the improved genetic algorithm was combined with the superior and inferior solution distance method to solve the genetic algorithm, and the corresponding objective function value was obtained after rounding. Finally, Pareto optimal solution set is selected by multi-objective decision, and the result of construction planning is obtained.

**Results:**

Based on multi-attribute decision analysis and taking three years as the construction cycle, the planning scheme of aged care institutions construction in each district of Nanjing in the next 15 years was proposed. In addition, considering the intelligent trend of future pension, the proportion of home-based pension is increasing year by year, and the model is promoted to meet the needs of diversified pension services. With the gradual increase of the elderly population in Nanjing, the proportion of intelligent home-based care has been increasing year by year since 2027, and the construction demand of aged care institutions has also increased. The number of construction at all levels rises gradually in each cycle, and the increase is more obvious after 2027.

**Conclusions:**

The layout and planning of aged care institutions proposed in this paper not only considers economic and environmental benefits, but also combines the current situation of aged care institutions in various districts of Nanjing. The model is reasonable and effective, and has practical application value. It will help China optimize the allocation of elderly care resources under the target of active aging, and scientifically and comprehensively deal with the problem of population aging.

## Introduction

The twenty-first century is an age of aging population. According to the Research Report on the Development Trend of Aging Population in China, China has entered the aging society in 1999, which is the earliest among the developing countries. As the country with the largest aging population in the world, China's aging is not only a problem of China itself, but also related to the process of global aging population, attracting worldwide attention. The aging of population has become a serious social problem in China at the present stage. The aging population is increasing year by year, and the decrease of the birth population makes the aging more obvious. According to the report, by October 2020, the elderly population aged 60 or above in China was 264 million, accounting for 18.70% of the total population. Thereinto, there are 191 million people aged 65 or above in China, accounting for 13.50% of the total population, and the number of elderly people aged 80 or above has reached 35.8 million.

China has a large population base, a fast decline in fertility rate, and an increasingly accelerated growth process of the elderly population, which may lead to a series of social problems such as "getting old before getting rich" and "getting old and poor" [1]. The state has timely made the overall strategic deployment of Mid- and Long-term National Plan for Actively Coping with Population Aging [2]. The "14th Five-Year Plan" period is an important strategic window of opportunity to actively deal with the aging of population. By the end of the "14th Five-year Plan", China is expected to enter the moderate aging stage from mild aging.

Due to the aging of fewer children and other factors caused by China's long-implemented family planning policy, the disabled elderly cannot receive comprehensive care at present. China urgently needs to develop socialized elderly care services and build a new elderly care system when long-term care needs are not met and families' capacity for long-term care is declining. However, there are many hidden dangers in the development of China's pension cause, such as the serious shortage of urban aged care institutions, the imperfect social security system, "getting old before getting rich" and other problems have become bottlenecks in the development of the pension industry. The Chinese government has gradually raised the issue of elderly care to a strategic level, putting forward such strategic requirements as "providing for the elderly", "giving priority to the development of community elderly care services" and "building a well-off society in an all-round way", which has made great progress in China's elderly care service system.

The aging rate of Jiangsu province is second only to that of Shanghai and Beijing, and it is also the province with the highest degree of aging. In the future, the aging population in Jiangsu will be accelerated. Jiangsu has entered into the "deep aging society", showing the typical characteristics of a large base of elderly population, a large proportion of the elderly, a high age, a straight rise in the elderly dependency ratio, prominent regional imbalance of aging. But it is still the strategic window period and opportunity period to deal with population aging actively. Therefore, in view of the major national needs of pension and elderly care, the optimal allocation of pension and elderly care resources under the goal of active aging is carried out, so as to put forward and formulate operational coping strategies and optimization plans from the perspective of Jiangsu province. It is an urgent issue for China to implement the requirements of "timely, scientific and comprehensive response to population aging", and is of great significance.

### Literature review

In terms of the prediction research on aging and disabled elderly, the prediction research on service demand in China started later than that in foreign countries, which mainly includes the prediction of social endowment service demand, long-term care cost, care labor demand and other aspects. Dai empirically analyzed the factors influencing the elderly's willingness to long-term care needs by using logit model [3]. On the basis of estimating the scale and variation of disability using Markov process, Hu et al. estimated the needs of elderly care services [4]. Zhang et al. adopted Sullivan's method to calculate the elderly's living care needs [5]. Based on the extended multi-state life table method, Jiang et al. analyzed the cost of daily life and end-of-life care for the elderly for the first time, and calculated the expected total cost of daily care [6]. Li calculated and analyzed the scale of long-term care funds for the disabled elderly in China [7]. Zhu et al. adopted the probabilistic transfer model and propensity value analysis to calculate the probability of the elderly's situation transfer, the time demand for care and its net difference, and forecast the demand for care labor [8]. Foreign research is relatively mature, in addition to the care of social assistance needs, also carried out macro and micro quantitative research on service demand. Wieland et al. simulated the disability elderly health deficit model and projected a mixed, modest cost for the elderly who received services for free [9]. Kingston et al. used a dynamic microsimulation model PACSim and Sullivan algorithm to predict the nursing needs of disabled elderly [10].

Social coping strategies for aging and disabled elderly mainly focus on pension mechanism, service resource allocation and other aspects of the research. Liao et al. found that with the growth of the elderly population, social security funds can hardly meet the needs of the elderly, and the commercial pension industry has attracted much attention from all walks of life [11]. Based on household interview survey, Sun et al. put forward suggestions on improving the quality and quantity of supply through effective allocation of medical and nursing resources [12]. Madero-Cabib et al. proposed to vigorously develop the pension industry and combine the insurance, real estate and pension industries to form an industrial chain for the elderly [13]. According to Caroline's research, although the government has formulated a series of policy measures to improve the efficiency of community care services, the main reason for the lack of service effectiveness is the allocation of funds [14]. By comparing the care policies of South Korea, Japan and Germany, Jong et al. believed that the government's establishment of social insurance could reduce the burden of care costs [15]. Duell et al. studied the long-term care system in the Netherlands and pointed out that service supply needs to be adapted to local conditions [16].

According to the above literature, the construction quality of pension institutions is not high, and the shortage of quantity is mainly due to the overall low service level and high operating cost. To be specific: (1) In terms of operation and management, traditional elderly care products and services, as public resources, mainly rely on the power of government departments to supply, and the overall level and level are low. The monopoly supply of public endowment institutions leads to the low efficiency of endowment resource allocation and directly causes the overall loss of social welfare. (2) In terms of the supply of elderly care products and services, although governments at all levels continue to increase the intensity of financial transfer payments, with the huge size of the elderly population, it is no longer possible to meet the development needs of the aging society by relying only on government-run public welfare nursing homes. Most pension institutions still stay in the traditional "raising", but ignore the needs of nursing care, cultural entertainment and other aspects. (3) In terms of professional service talents, affected by factors such as professional reputation and labor remuneration, the number of employees willing to devote themselves to pension institutions is insufficient at present, and professional talents are scarce, so the professionalization model has not been formed. Most employees lack professional knowledge background and professional skills training, which will restrict the long-term development of the elderly care industry. (4) In terms of socialization, industrialization and scale, due to the limited funds invested by the government and the large number of public pension institutions across the country, all kinds of social welfare institutions that have been built generally have problems of small scale, low grade, poor conditions, and low quality of management and service. Therefore, the current domestic endowment institution construction of socialization, industrialization, scale is low. (5) In terms of the utilization rate of pension institutions, due to regional overall planning, the distribution of the elderly population in the city, the educational level and economic level of the elderly, the utilization rate of beds is not balanced. While some institutions are "hard to get a bed", others are "empty". In short, when considering the above factors, the previous literature usually takes economic benefits as the starting point to study the supply and demand situation of institutional elderly care from the number of institutions, number of beds, occupancy rate, institutional configuration and other aspects, so as to build a single objective optimization model with economic benefit maximization as the objective function. On the basis of previous studies, this paper comprehensively considers the maximization of economic benefits and social benefits, so as to construct a multi-objective optimization model, and the construction strategy is more comprehensive and more realistic.

### Present situation of nursing service for the disabled elderly

In Canada, long-term care services are jointly completed by government staff, community agency staff and home care staff [17], including home care, institutional care and hospice care. Home care means that the elderly can enjoy life and spiritual care services at home. Institutional care is the comprehensive physical and psychological care of the elderly in public or private aged care institutions. Hospice care refers to the care provided to the terminally ill individual and their family members. The focus of care is to enable the cared for person to live comfortably, stress-free or painless in the limited life time [18]. The American model of care is a professional care service for the elderly who cannot take care of themselves physically and mentally. Long-term care services combined with voluntary and compulsory care should be adopted, including routine family care, day care for the elderly, hospice care, etc. [19]. Germany has a relatively perfect welfare system in the world, and old-age security is an important part of it. In Germany, the rich and sound insurance system includes state-stipulated endowment insurance, workplace endowment insurance, individual life insurance, social welfare and assistance, etc. [20]. In addition to the insurance system, there are also various modes of mutual assistance for the aged, including mutual assistance between the elderly; mutual assistance between the elderly and single-parent families; joint assistance between the elderly with care needs and the students with accommodation needs [21].

China's old-age security has been supporting the vulnerable and disadvantaged groups, the " three non-personnel " and the "households enjoying the five guarantees" in urban and rural areas, and finally reaching all the people. In recent years, the government has issued a number of policy documents to promote the socialization of pension services, calling for social attention to aged care institutions. However, the long cycle, slow return, large investment, non-profit and other characteristics limit the development of pension service, resulting in a shortage of pension service supply [22, 23]. From the original intention of the construction of the old-age care service system, self-care elderly people generally receive home-care services at home, while semi-self-care elderly people enjoy old-age care services provided by the community at home. Only the very old and disabled should be the main target of nursing homes. Compared with the self-care elderly, the disabled elderly are obviously less capable of self-care and need assistance from others in daily life. At the same time, disabled elderly people have higher requirements for professional medical care. At present, most pension institutions can achieve the "integration of medical care and nursing", and pension institutions can reasonably use existing medical resources to meet the medical care needs of the disabled elderly. Therefore, compared with family care and community care, institutional care has more advantages in providing professional care and physical and mental counseling for the disabled elderly.

However, at present, the family pension is the main pension mode for the disabled elderly in China. The "421" family mode makes the children of the disabled elderly bear great pension pressure, resulting in the disabled elderly often cannot get good care. China's community elderly care service has been developing since the late 1980s. Compared with western developed countries, it develops slowly, but the overall construction has begun to take shape [24]. By the end of 2020, there were 329,000 care institutions and facilities for the elderly nationwide, an increase of 125,000 or 61.3% over the previous year. The number of beds for the elderly totaled 8.21 million, an increase of 0.46 million or 5.9% over the previous year. Among them, 38,000 nursing homes were registered, an increase of 11.0%, and 4.882 million beds were registered, an increase of 11.3%. There were 291,000 community care institutions and facilities, and 3.328 million community care beds [25].

### Analysis of the current situation of China's aged care institutions

China has entered a serious aging period. The aging population is increasing year by year, and the decrease of the birth population makes the aging more obvious. The number of pension groups continues to increase, increasing demand for pension. Under the support of the government and society, the number of aged care institutions has increased, but by comparison, the number is obviously not enough to meet the needs of pension. The resources of aged care institutions are also relatively poor, and the related infrastructure is not perfect, such as the shortage of beds. Supply contradiction is more and more obvious, supply structure is also serious imbalance. At the same time, the supply of professional talents related to old-age care is insufficient, and social service talents in old-age care institutions are insufficient, presenting problems such as low quantity and poor quality. Most aged care institutions have simple service projects and lack of professional medical services, which cannot meet the needs of the disabled elderly [8]. The proportion of professional nursing staff in nursing institutions is very low, and the high cost of care discourages disabled elderly people from entering professional nursing institutions. The high cost also forces nursing homes to choose healthy elderly people with lower care costs. According to the "National Study on the Status of disabled Elderly in Urban and Rural areas", nearly half of the institutions for the elderly have made it clear that they are unwilling to accept disabled elderly. It can be seen that China has a long way to go to solve the pension problem. If the disabled elderly can be satisfied in the aspects of physiology, psychology and health, and family pressure can be relieved, the government and relevant departments need to introduce policies, guide the work direction, strengthen publicity and obtain social support [9]. Under the background of aging with Chinese characteristics, pension institutions are still the first choice for the disabled elderly in the future, so the construction of pension institutions needs to be solved urgently.

By the end of 2020, the number of registered elderly over 60 years old in Nanjing has reached 1.581 million, accounting for 22% of the city's registered population. Meanwhile, the elderly population is increasing by 40,000 to 50,000 each year. Among them, there are 244,000 registered seniors over 80 years old, accounting for 15.4% of the registered elderly population. There are more than 115,000 disabled or semi-disabled people in the city, indicating that Nanjing has entered a "deep aging society". In 2019, the document issued by the Jiangsu Provincial Civil Affairs Department announced that the establishment license of aged care institutions would no longer be implemented, and only the record work of aged care institutions would be done. Therefore, timely construction of aged care institutions will become a problem that the pension service industry must focus on. Nanjing is in the leading position in old-age services nationwide, and was listed as "National pilot city for comprehensive reform of old-age services" in 2014. However, from the overall situation of Nanjing, social aged care institutions are seriously insufficient, and pension services fail to fully meet the pension needs [10]. Until 2019, there are only 245 aged care institutions in Nanjing, and the proportion of the elderly population in Nanjing will continue to increase year by year in the coming years. The characteristics of the elderly population, such as "fast growth, long longevity, empty-nest and disability", are more obvious, and various new pension methods are gradually emerging. Therefore, it can be seen that meeting the needs of diversified pension services and building aged care institutions will become one of the urgent livelihood issues in China [11].

### The significance of pension institution construction

At present, domestic is given priority to public pension institutions. Nevertheless, due to the "low price and high quality" of public pension institutions, there is a shortage of beds. In comparison with private pension institutions with higher prices, many elderly people will choose to keep on waiting for beds in public pension institutions, which is not conducive to the solution of the pension problem. In addition, public pension institutions are more dependent on the government's financial input. If they continue to be dominated by public pension institutions, it will bring serious burden to the public finance. As a result, referring to the experience of foreign and domestic pension institutions, the government should transform its function from the undertaker to the supervisor of pension institutions. The government could consider handing over the market-oriented pension services to social organizations and private sector. Meanwhile, it is necessary to give full play to and mobilize the enthusiasm of non-profit organizations to increase the supply of institutional elderly care services. While the government only needs to supervise, guide and provide necessary funding to private pension institutions. On the one hand, it can reduce the government's management cost, on the other hand, it can effectively improve the efficiency of supply of pension services, and mobilize the enthusiasm of all sectors of society to participate in pension services. Therefore, the construction of pension institutions proposed in this paper is biased toward the construction of private pension institutions.

Form pension service system for the construction of the original intention, community pension institutions should be mainly aimed at the semi-self-care elderly to provide some medical and life help for their home care. However, private pension institutions should be used to adopt the elderly, disabled and mentally disabled with the support of the government, and provide comprehensive help for them. From the perspective of pension policy, community pension policy points out that community service network suitable for the life and nursing of the elderly should be gradually established. At the same time, community support for the elderly will also be the key and core of improving pension services in the future. Nevertheless, due to the limitation of the number and time of service personnel, the community pension institutions can only alleviate the living problems of the elderly to a certain extent. Institutions pension, by contrast, as a special place for the elderly, does a better job in life care than community endowment. In addition, from the perspective of pension service content, community and institutional care for the elderly currently will provide daily care, leisure and entertainment activities and spiritual care services. The services provided by the community endowment will be more diversified and targeted, which can satisfy the individual needs of the elderly. While institutional care is mainly for the care of daily life, in other aspects of the service is limited. In conclusion, although there are differences between the community pension and institutional pension, as long as they distribute their respective functions, coordinate and complement each other, they can make full use of pension resources and provide all-round services for the elderly, so as to better solve the problem of pension.

### Organization and framework

In order to meet the pension needs of Nanjing in the next 15 years, this paper establishes a multi-objective planning model for the construction and layout of aged care institutions based on the principle of having as many beds as possible per 1,000 registered elderly under the premise of considering both economic benefits and social costs. Considering that the social cost needs to be reduced while the economic benefit is satisfied, and the number of nursing homes can only be integer, the multi-objective genetic algorithm is improved with the idea of integer programming, and the Pareto optimal solution set satisfying the conditions is obtained through multiple optimization. Finally, based on the theory of multi-attribute decision analysis, the planning scheme of aged care institutions construction in the next 15 years in each district of Nanjing is given.

## Methods

### Model objective function and constraint conditions

In 2019, Nanjing Municipal Government issued several Opinions on Improving the Service Quality of Nursing Homes in Nanjing (Trial), pointing out the current and future development needs of nursing homes. From the beginning of 2021 to the end of 2035, in accordance with the principle that the higher the level of aged care institutions, the higher the charge, the planning of aged care institutions should be considered to meet the needs of the elderly with low, lower, middle, upper and high disposable income in Nanjing.

The allocation of aged care institutions should consider at least two aspects of economic benefits and social costs, among which the government allocation of aged care institutions should take into account both fairness and efficiency, in line with the principle of "maximum fairness and maximum allocation efficiency". It is expected that the elderly population in Nanjing will reach 2.56 million in 2035, and according to the “Opinions on Improving the Service Quality of Nursing Homes in Nanjing (Trial)” issued by Nanjing Municipal Government at the regular press conference held on January 17, 2019, there were 39 beds for every 1,000 elderly people in Nanjing by the end of 2018. By 2020, there will be 45 beds for every 1,000 elderly people in the city. If construction grows at this rate, the city is expected to have 90 beds for every 1,000 elderly people by 2035. However, due to the impact of many emergencies or uncertainties, such as the COVID-19 outbreak from the end of 2019, the speed of construction has not increased steadily as expected. Consequently, it is required that there should be 60 beds for every thousand registered elderly in Nanjing by December 2035, and the construction planning scheme for the next 15 years should be provided with a construction period of three years.

The purpose of this paper is to meet the needs of nursing home construction at the same time to obtain the maximum economic and social benefits. Under normal circumstances, the benefits are mainly embodied in the material and spiritual two levels. However, it is difficult to quantify the benefits at the spiritual level, such as spiritual morality, social atmosphere, ideology and culture, so this paper only considers the benefits at the material level, which are usually achieved by reducing costs. In this paper, accommodation fees, taxes and other expenses for the inflow of funds for the nursing home are defined as economic benefits, and depreciation fees, utilities and transportation fees and other expenses for the outflow of funds for the nursing home are defined as social costs. Therefore, reducing social costs as much as possible is to realize the maximization of social benefits, and the two have a negative relationship. Therefore, the economic benefits and social costs of aged care institutions are taken as the objective function of the model. The mathematical model of the objective function is described as follows:

**Objective 1:** Economic benefit *F*_*1*_.

The economic benefit *F*_*1*_ in this paper refers to the profit derived from accommodation fees, government subsidies, tax incentives, etc. The average income of aged care institutions at all levels is used to measure economic benefit. The specific calculation formula is as follows:

1 $$F_1=\max\;\sum_{i=1}^M\;\sum_{j=1}^N\;b_{\mathit i\mathit j}x_{\mathit i\mathit j}$$

**Objective 2:** Social cost *F*_*2*_.

The social cost *F*_*2*_ includes rental costs, wages of employed service personnel, depreciation costs of facilities, water and electricity costs, transportation costs, food costs, etc., that is, as social costs. Considering that the more aged care institutions there are, the more they can meet pension needs and bring more benefits, the social costs will also increase. Therefore, how to achieve the maximum economic benefit at the minimum social cost is the focus. It is proposed to measure the social cost with the area standard of each grade of aged care institutions. The specific calculation formula is as follows:

2 $$F_2=\min\overset M{\underset{i=1}{\sum\;}}\sum_{j=1}^Na_{\mathit i\mathit j}x_{\mathit i\mathit j}$$

where *x*_*ij*_, *a*_*ij*_ and *b*_*ij*_ are the construction number, construction cost coefficient and income coefficient of the *j*-th level aged care institutions in the *i*-th cycle respectively. *M* is the total cycle, and each cycle is bounded by three years, with a total of five cycles; *N* is the number of grades, *N* = *5*, from low to high, there are five grades A-5A.

The less the cost is paid, the more funds can be saved to improve the facilities and quality of nursing homes, and the government can be encouraged to build more nursing homes so as to solve the pension problems of more elderly people. *F*_*1*_ is revenue and *F*_*2*_ is expenditure, and the two objectives interfere with each other. The increase of *F*_*1*_ requires an increase in the number or scale of pension institutions, which is bound to lead to the growth of *F*_*2*_. The purpose of this paper is to find a compromise solution that can maximize *F*_*1*_ and minimize *F*_*2*_ at the same time, so as to maximize the benefit.

At the same time, the model also needs to meet the following six constraints, and the mathematical model of the constraints is described as follows:

1. According to the requirement that there should be 60 beds for every 1,000 registered elderly in Nanjing city by December 2035, the restrictions are as follows:

3 $$\sum_{i=1}^N{\mathrm d}_{0\mathrm j}+\overset M{\underset{i=1}{\sum\;}}\sum_{j=1}^Nc_{\mathrm{ij}}x_{\mathrm{ij}}\geq0.06\;\ast\;P_{2035}$$

(2) According to the elderly demand of Nanjing with low, lower, middle, upper and higher disposable income, there are five constraints as follows:

4 $$d_{0\mathrm k}+\sum_{\mathrm i=1}^Mc_{\mathrm{ik}}x_{\mathrm{ik}}\geq p_{\mathrm k}\left(\overset M{\underset{\mathrm i=1}{\sum\mathit\;}}\sum_{\mathrm j=1}^{\mathrm N}{\mathrm c}_{\mathrm{ij}}x_{\mathrm{ij}}+\overset N{\underset{\mathrm j}{\sum d_{\mathrm j}}}\right),\;k=1,2,3,4,5$$

where, *d*_0*j*_ is the current number of beds at the *j*-th level; *c*_*ij*_ is the bed coefficient of the *j*-th level nursing home in the *i*-th cycle, which can be replaced by the mean value of the current data of nursing home at all levels. P_2035_ is the number of elderly population in 2035, and *p*_*j*_ is the minimum proportion of the number of beds at the *j*-th level in the total number of beds [12].

In the objective functions (such as economic benefit) and constraints, other factors such as income and population are taken into account. People with different incomes will choose beds of different levels, that is, people with higher incomes are more inclined to choose beds of higher levels. This population is relatively small, so relatively few beds are being built, and vice versa. The population pressure is also distributed in proportion to income in the number of beds built at each level.

Considering the rationality and feasibility of the model, this paper makes the following basic assumptions for the model:

#### Assumption 1

It is assumed that the elderly in each region receive old-age care services in the region where they live for a long time, and there is no cross-district or personnel flow.

#### Assumption 2

The influence of the change of Nanjing administrative region in recent years on the data and results of this paper is not considered.

#### Assumption 3

It is assumed that the elderly's choice of each level of nursing institution corresponds to their level of disposable income.

The model established in this paper has certain universality, which can change relevant parameters according to subjective needs, such as the initial number of beds, the estimated number of beds per thousand elderly people, cost coefficient, etc., so that the optimal decision scheme can be planned after recalculation.

### Improved multi-objective genetic algorithm based on integer programming

**Fig. 1:**
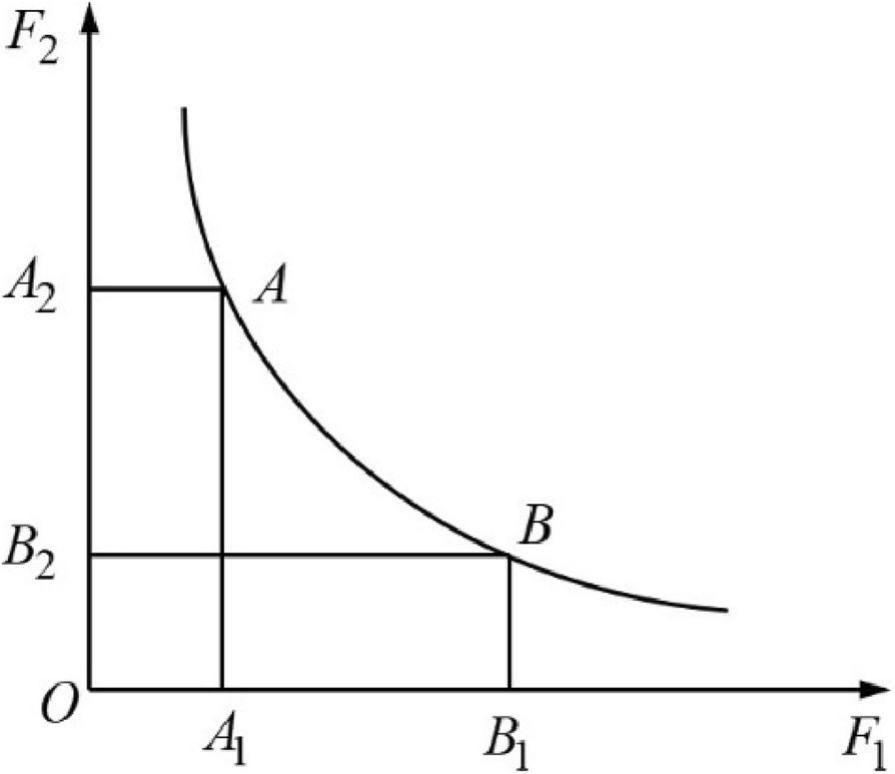
Pareto optimal solution set

The allocation of aged care institutions needs to take into account both economic benefits and social costs. In the modeling, we hope to maximize both economic benefits and social benefits, which is a multi-objective optimization problem. However, social benefits can only be measured by minimizing social costs. Because the two objective functions often interfere with each other and influence each other, it is necessary to reduce social costs while satisfying economic benefits, and vice versa. Therefore, the solution of the model is a series of non-inferior solutions, namely Pareto optimal solutions. The solution to a multi-objective problem is in the form of a Pareto optimal solution set, a solution in which no objective can be improved without reducing at least one of the other objectives. The set composed of Pareto optimal solutions is often the Pareto frontier, as shown in Fig. 1.

**Fig. 2:**
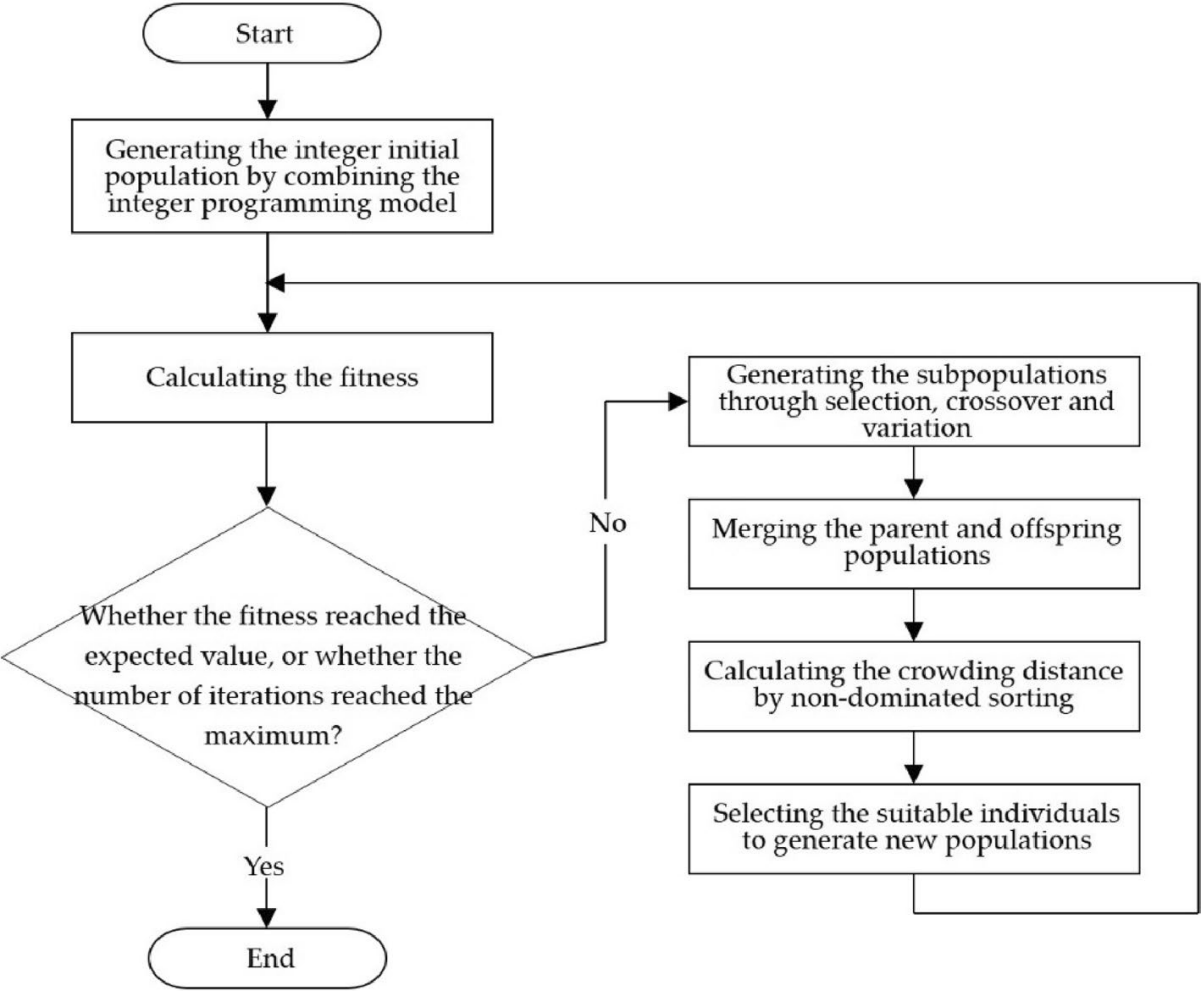
Flow chart of multi-objective integer programming algorithm

Since the number of nursing homes to be built must be integers, it is a multi-objective integer programming problem. But multi-objective genetic algorithm cannot solve the integer programming problem in previous literatures. Therefore, it is considered that the integer programming model is combined with the multi-objective genetic algorithm by taking the integral function, and the global optimal solution is realized through multiple optimization searches, i.e., multi-objective integer programming [13, 14]. Among them, the method of roulette is adopted in the selection step of genetic algorithm. The specific algorithm flow is shown in Fig. 2, and the basic steps are as follows:

**Step 1:** Generate the initial population. N integer samples were randomly generated by integer programming and the maximum evolution algebra of the population was set.

**Step 2:** If the individual fitness has reached the threshold, or its value no longer has an upward trend, or the number of iterations of the algorithm has reached the pre-set algebra, stop the operation and get the Pareto optimal solution set. Otherwise, repeat the following steps:

1. Through selection, crossover and mutation operation, the offspring population is generated and merged with the parent.
2. The order values of individuals in the combined population were calculated and the non-dominant solution was screened. The crowding distance between individuals was calculated. The larger the crowding distance was, the better the population diversity was.
3. Select suitable individuals as the new parent population.

**Step 3:** Draw the image and terminate the operation.

Firstly, genetic algorithm (GA) can avoid falling into local optimum through mutation mechanism, and has strong searching ability. Secondly, by introducing the idea of probability in natural selection, individual selection has randomness. Finally, the application of genetic algorithm is mature and extensible. There are many improved methods and it can be used in combination with other algorithms. Compared with the traditional optimization algorithm, the optimization result of the genetic algorithm is independent of the initial conditions, and it can find the global optimal solution of the optimization problem, which has strong robustness. At the same time, the binary encoding and decoding method of genetic algorithm can be well adapted to the discrete decision variable optimization problem in which the number of pension institutions must be integer.

### Multi-objective decision analysis

Economic benefit and social cost in the model of aged care institutions layout are two opposite goals. The more aged care institutions, the higher the economic benefits. But at the same time social costs will rise, so the two goals are at odds with each other. A series of optimal solution sets satisfying the requirements can be obtained by using genetic algorithm, and the two target values corresponding to each set of solutions are different. Therefore, it is necessary to find an appropriate method to select the solution that can best meet the needs of decision makers in this series of non-inferior solutions, that is, the construction scheme of aged care institutions. Therefore, the pros and cons solution distance method in multi-objective decision making is proposed [15].

Multi-objective optimization problems have multiple objectives and each objective function is contradictory, that is, the approach of one objective function to the optimal value will make another objective function away from the optimal value. Therefore, the solution form of multi-objective optimization problem is a set of non-inferior solutions, so it is very important to select and determine the optimal solution so that the two objectives are as close to the optimal value as possible. Multi-objective decision making methods mainly include Hierarchical Sequence Method, Analytic Hierarchy Process (AHP), Reordering Method, and Advantages and Disadvantages Solution Distance Method (TOPSIS) [16]. The traditional TOPSIS method has the problem of reverse order caused by the change of ranking due to the change of scheme, and the failure of Euclidean distance measurement due to the influence of correlation between index data. The inverse order problem can be solved by the absolute ideal solution method, and the weighted Mahalanobis distance can replace the Euclidean distance to solve the problem affected by the correlation between index data.

It is mainly considered to adopt the TOPSIS method. Compared with other evaluation models, this method has no strict restrictions on data distribution and sample size, and the data calculation is simple and easy. The selection of the maximum and minimum value in the group and the calculation of the gap between each index and the optimal value and the worst value are very objective. And the importance of each goal can be adjusted according to the subjective preference of the decision maker. So we can sort and filter according to the actual needs and get the most satisfactory solution. Its basic principle and operation steps are as follows:

The Pareto solution set is expressed in the form of matrix, where *S*_*1*_*, S*_*2*_*, S*_*m*_ represents the Pareto solution set (i.e. the scheduling scheme to be selected), *O*_*1*_*, O*_*2*_*,⋯, O*_*n*_ is the objective function, and *f*_*ij*_ represents the *j*-th objective function *O*_*j*_ corresponding to the *i*-th Pareto solution *S*_*i*_, as shown in Table 1:

**Table 1:** The matrix form of Pareto solution set

	*O*_*1*_	*O*_*2*_	$\cdots$	*O*_*n*_
*S*_*1*_	*f*_*11*_	*f*_*12*_	$\cdots$	*f*_*1n*_
*S*_*2*_	*f*_*21*_	*f*_*22*_	$\cdots$	*f*_*2n*_
$\vdots$	$\vdots$	$\vdots$	$\ddots$	$\vdots$
*S*_*m*_	*f*_*m1*_	*f*_*m2*_	$\cdots$	*f*_*mn*_

Step 1: Calculate the standardized decision matrix ${n}_{ij}=\frac{{f}_{ij}}{\sqrt{\sum_{i=1}^{m}{f}_{ij}^{2}}}, i=1, \cdots, m, j=1, \cdots , n$, according to *f*_*ij*_.

Step 2: Calculate the weight ${v}_{ij}={\omega }_{j}{n}_{ij}, i=1, \cdots, m, j=1, \cdots , n$ of the normalized matrix *v*_ij_ by *n*_*ij*_, where ω_j_ > 0 is the weight of the *j*-th objective function, and ${\sum }_{j=1}^{n}{\omega }_{j}=1$.

Step 3: The ideal solution *A*^*+*^ and the non-ideal solution *A*^*-*^ are determined as follows:

5 $$A^{\mathit+}\mathit=\mathit{\left\{{v_1^+,\;\cdots\;v_n^+}\right\}}\mathit=\mathit{\left\{{\underset j{min}\left\{v_{ij}\left|i\in I\right.\right\},\;\underset j{min}{\left\{v_{ij}\left|i\in I\right.\right\}}}\right\}}$$

6 $$A^{\mathit-}\mathit=\left\{v_{\mathit1}^{\mathit-}\mathit,\mathit\;\mathit\cdots\mathit\;v_n^{\mathit-}\right\}\mathit=\left\{\underset j{man}\left\{v_{ij}\left|i\mathit\in I\right.\right\}\mathit,\mathit\;\underset j{man}\left\{v_{ij}\left|i\mathit\in I\right.\right\}\right\}$$

where *I* and *J* represent economic cost and environmental cost respectively.

Step 4: Based on ideal points and non-ideal points, the separation measures ${d}_{i}^{+}$ and ${d}_{i}^{-}$ are generated as follows:

7 $$d_i^+=\sqrt{\begin{array}{cc}\sum_{j=1}^n\left(v_{ij}-v_j^+\right)^2\end{array}}\left(i=1,\;\cdots\;,\;m\right)$$

8 $$d_i^-=\sqrt{\begin{array}{cc}\sum_{j=1}^n\left(v_{ij}-v_j^-\right)^2\end{array}}\left(i=1,\;\cdots\;,\;m\right)$$

Step 5: After calculating the relative distance ${R}_{i}=\frac{{d}_{i}^{-}}{{d}_{i}^{+}+{d}_{i}^{-}}, i=1, \cdots, m$ from the ideal point, the corresponding Pareto solution when R_i_ (R_i_ ≤ 1) is the largest is the final decision solution.

In this paper, the elements of the Pareto solution set are deterministic and there is no change of the scheme, so it is not affected by the inversion problem. Different elements are independent and there is no correlation between them, so Euclidean distance calculation is effective. And through the subjective method to determine the weight that economic benefits and social costs are equally important. The problem of reverse order is caused by the creation and reduction of evaluation objects. However, the solution set obtained by the optimization algorithm in this paper contains a certain number of variables. And each variable is independent of each other, there is no correlation effect. Therefore, the traditional TOPSIS method is directly used to screen the Pareto solution set without considering the limitations of TOPSIS method. In addition, this paper considers that economic benefits and social costs are equally important, so the weights of the two are determined by subjective methods, and both are set at 0.5.

## Results

### Description of model parameter selection

According to the Rating Standards for Nursing Homes in Nanjing, nursing homes are divided into 5 levels, including basic conditions, hardware facilities, internal management, service provision and social evaluation. A total of 96 factors, such as building area, single bed area, single room area, double room area, bathroom and laundry facilities, were subdivided and the relevant score of each index was calculated. The final score above 800 was considered as the qualified score of the relevant grade of nursing home. According to the evaluation results of aged care institutions issued by Nanjing Civil Affairs Bureau in 2019, the distribution of aged care institutions of all levels in each region can be sorted out, as shown in Table 2:

**Table 2:** The distribution of aged care institutions in different districts of Nanjing

Number of institutions	AAAAA	AAAA	AAA	AA	A
Xuanwu	0	2	8	2	0
Qinhuai	0	6	8	7	5
Gulou	0	7	15	3	5
Liuhe	0	1	3	1	1
Jianye	1	2	4	0	2
Yuhuatai	0	2	10	2	4
Qixia	2	4	1	9	7
Pukou	1	1	3	6	1
Jiangning	2	6	8	2	8
Lishui	0	1	4	1	1
Gaochun	0	1	0	0	0

In addition, information such as nearly 30 aged care institutions, charging standards and number of beds in Nanjing were collected, covering all levels of aged care institutions in all regions of Nanjing, among which the number of institutions from AAAAA grade to A grade was 5, 10, 7, 3 and 3 respectively. According to the "Nanjing Statistical Yearbook—People's Life", from 2010 to 2012, the percentage of households grouped by each income level and the corresponding disposable income data of each group are given (as shown in Table 3). As the year changes, so does the disposable income criteria by which groups are judged, and the prescribed criteria vary according to the year. Accordingly, the per capita income and expenditure level of urban households in the city from 2010 to 2012 was collected: the lowest income and low income households accounted for 10% respectively, the lower middle income households, middle income households and upper middle income households accounted for 20% respectively, and the high income households and the highest income households accounted for 10% respectively. It can be inferred that the population with low, lower, middle, upper and high disposable incomes respectively account for 20% in Nanjing. Therefore, in order to meet the needs of aged care institutions at different economic levels and maintain a certain flexibility of the market, the minimum proportion *p*_*j*_ of the number of beds in the total number of beds is uniformly selected as 15%. That is, the number of aged care institutions at each level should reach at least 15% of the total number. In general, 75% of aged care institutions can meet the needs of various consumption levels.

**Table 3:** Classification of urban households in Nanjing by per capita income and expenditure level (2010–2012)

Year	Index	Total survey households in the city	Group by income level						
			Lowest income households (10%)	Low income households (10%)	Lower middle income households (20%)	Middle income households (20%)	Upper middle income households (20%)	High income households (10%)	Highest income households (10%)
2010	A (Household)	800	80	80	160	160	160	80	80
	B (Yuan)	28,312	9311	14,174	18,652	24,264	34,025	46,742	72,481
2011	A (Household)	800	80	80	160	160	160	80	80
	B (Yuan)	32,200	11,329	17,014	22,371	28,693	38,585	50,557	76,384
2012	A (Household)	800	80	80	160	160	160	80	80
	B (Yuan)	36,322	13,715	20,279	25,770	32,412	42,943	54,883	83,954

Index A: Number of households under Investigation (Household)

Index B: Disposable income (Yuan)

Based on the above criteria, this paper sorted out the distribution data of pension institutions of various levels in various regions of Nanjing in 2019. The initial number of beds was set according to a certain proportion by means of sampling survey combined with the information of beds and charging standards provided by institutions of various levels. By means of sampling survey, the charging standard and the number of beds of aged care institutions at all levels can be calculated, that is, the *b*_*ij*_ and *c*_*ij*_ coefficients in the layout model of aged care institutions. The minimum construction area of nursing homes at all levels can be known according to the "Rating Assessment Standard for Nursing Homes in Nanjing (Trial)" issued by Nanjing Municipal Civil Affairs Bureau in 2019. 1.5 times of the minimum standard is used to describe the construction cost of aged care institutions of all levels, that is, the coefficient *a*_*ij*_ in the layout model of aged care institutions. The initial number of beds *d*_*j*_ in each district can be inferred by using the data of *c*_*ij*_ and Table 2. Specific model parameters are listed in Table 4, as follows.

**Table 4:** The selection of model parameters

Parameter	AAAAA	AAAA	AAA	AA	A
*a*_*ij*_	11,250	7200	3600	1875	375
*bij*	7916	5480	2771.428571	2333.333333	750
*c*_*ij*_	777.6	265.4	168.1428571	74.33333333	35
*d*_*j*_	AAAAA	AAAA	AAA	AA	A
Xuanwu	0	532	1352	150	0
Qinhuai	0	1596	1352	525	175
Gulou	0	1862	2535	225	175
Liuhe	0	266	507	75	35
Jianye	778	532	676	0	70
Yuhuatai	0	532	1690	150	140
Qixia	1556	1064	169	675	245
Pukou	778	266	507	450	35
Jiangning	1556	1596	1352	150	280
Lishui	0	266	676	75	35
Gaochun	0	266	0	0	0

### Construction planning scheme of each district of Nanjing in the next 15 years

In this paper, we hope to obtain maximum economic benefit with minimum social cost, which is a multi-objective optimization problem. However, the result of a multi-objective problem is usually a Pareto optimal solution set form in which no objective can be improved without reducing at least one other objective. Therefore, the objective function should be maximized or minimized simultaneously. In the actual solution, this paper takes the negative value of economic benefit, so as to minimize the negative value of social cost and economic benefit at the same time.

**Fig. 3:**
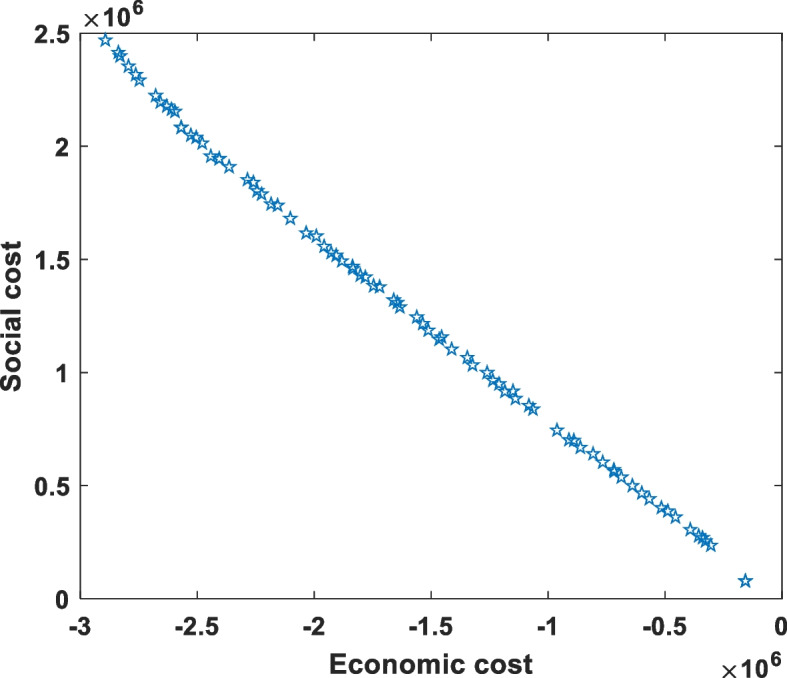
Pareto optimal solution set in Xuanwu

According to the multi-objective optimization model established above, the improved genetic algorithm and the superior and inferior solution distance method are combined. With the help of MATLAB software, the model is solved by genetic algorithm first, the parameters of the genetic algorithm are set as 'paretoFraction',0.8,'populationsize',100,'generations',500, to obtain 80 sets of solutions under each Pareto optimal solution set, and the solution result is rounded, and then the value of the corresponding objective function is calculated. Then the Pareto optimal solution set is selected by multi-objective decision making method and the result of construction planning is obtained. When selecting the optimal solution with multiple objectives, the corresponding weights of *F*_*1*_ and *F*_*2*_ are 0.5 and 0.5 (the actual selection of weights can be determined according to the goal needs of decision-makers). Due to the limitation of space, we only give a group of optimal solutions after the decision. That is, the planned number of aged care institutions in each district of Nanjing in the next 15 years are listed in Tables 5, 6, 7, 8, 9, 10, 11, 12, 13, 14, 15, as follows. Meanwhile, taking the Xuanwu district as an example, the results of Pareto optimal solution set are given as shown in Fig. 3.

**Table 5:** Number of aged care institutions proposed in Xuanwu

Year	A	AA	AAA	AAAA	AAAAA
2021–2023	211	98	2	1	1
2024–2026	14	63	1	1	1
2027–2029	67	74	1	1	1
2030–2032	3	2	1	2	1
2033–2035	90	108	2	1	1

**Table 6:** Number of aged care institutions proposed in Qinhuai

Year	A	AA	AAA	AAAA	AAAAA
2021–2023	116	42	5	9	1
2024–2026	130	16	9	4	2
2027–2029	1	326	20	6	5
2030–2032	4	168	6	10	1
2033–2035	1	20	4	8	4

**Table 7:** Number of aged care institutions proposed in Gulou

Year	A	AA	AAA	AAAA	AAAAA
2021–2023	296	195	1	2	1
2024–2026	7	77	2	1	1
2027–2029	26	180	4	2	1
2030–2032	152	178	2	2	1
2033–2035	2	18	11	4	1

**Table 8:** Number of aged care institutions proposed in Liuhe

Year	A	AA	AAA	AAAA	AAAAA
2021–2023	380	17	4	2	1
2024–2026	213	24	3	2	1
2027–2029	10	19	2	3	1
2030–2032	195	45	4	2	1
2033–2035	10	61	1	1	1

**Table 9:** Number of aged care institutions proposed in Jianye

Year	A	AA	AAA	AAAA	AAAAA
2021–2023	54	0	0	0	0
2024–2026	20	0	0	0	0
2027–2029	21	0	0	0	0
2030–2032	6	0	0	0	0
2033–2035	1	0	0	0	0

**Table 10:** Number of aged care institutions proposed in Yuhuatai

Year	A	AA	AAA	AAAA	AAAAA
2021–2023	18	0	0	0	0
2024–2026	16	0	0	0	0
2027–2029	12	0	0	0	0
2030–2032	1	0	0	0	0
2033–2035	0	0	0	0	0

**Table 11:** Number of aged care institutions proposed in Qixia

Year	A	AA	AAA	AAAA	AAAAA
2021–2023	45	5	3	2	1
2024–2026	46	3	7	2	1
2027–2029	4	0	3	2	1
2030–2032	0	11	3	1	1
2033–2035	0	2	1	2	1

**Table 12:** Number of aged care institutions proposed in Pukou

Year	A	AA	AAA	AAAA	AAAAA
2021–2023	32	6	1	0	0
2024–2026	51	0	0	0	0
2027–2029	46	0	0	0	0
2030–2032	43	0	0	0	0
2033–2035	36	0	0	0	0

**Table 13:** Number of aged care institutions proposed in Jiangning

Year	A	AA	AAA	AAAA	AAAAA
2021–2023	145	0	0	0	0
2024–2026	111	0	0	0	0
2027–2029	25	0	0	0	0
2030–2032	1	0	0	0	0
2033–2035	0	0	0	0	0

**Table 14:** Number of aged care institutions proposed in Lishui

Year	A	AA	AAA	AAAA	AAAAA
2021–2023	98	0	0	0	0
2024–2026	80	0	0	0	0
2027–2029	91	0	0	0	0
2030–2032	33	0	0	0	0
2033–2035	12	0	0	0	0

**Table 15:** Number of aged care institutions proposed in Gaochun

Year	A	AA	AAA	AAAA	AAAAA
2021–2023	107	57	6	2	2
2024–2026	210	88	11	4	2
2027–2029	3	88	5	2	2
2030–2032	6	9	16	6	2
2033–2035	5	36	7	5	2

### Planning of aged care institutions construction for smart community

Artificial intelligence has many advantages in elderly care, which is the trend of the future development of society. Artificial intelligence can carry out real-time health monitoring and security monitoring to avoid accidents among empty nesters. For the disabled and semi-disabled elderly, artificial intelligence can cope with the daily chores only through voice recognition, and avoid friction and conflicts between the elderly and caregivers, so as to provide the elderly with higher quality care. In addition, the development of artificial intelligence can help alleviate the shortage of nursing institutions and nursing professionals.

Considering that intelligent robots will replace human services from 2027 and the proportion of elderly care will increase year by year, the objective function and constraint conditions of the institutional construction layout model are adjusted. The labor cost coefficient should be reduced, and the bed requirement should be increased per 1,000 registered elderly. According to the number of elderly population in Nanjing in the next 15 years, the bed requirements are allocated to each cycle and the corresponding constraints are adjusted. Taking The Xuanwu District of Nanjing city as an example, the construction plan of the elderly care institution in the next 15 years is formed in Xuanwu District, so that it cannot only meet the actual demand, but also consider the economic benefits and social costs under the background of robot replacing human services.

It is assumed that intelligent robots can replace human services from 2027, and the proportion of home care in intelligent communities increases year by year [17]. After human services are replaced by intelligent robots, the income of nursing workers in aged care institutions will decrease. In addition, the proportion of home-based elderly care increases year by year, indicating that the demand for elderly care increases, and the number of beds for elderly care should increase to more than 60 per 1,000 registered residents. Different from the previous layout model, the first two cycles are the same from 2021–2026, but the economic returns in the target function need to change from 2027. The number of beds per 1,000 registered elderly people also needs to increase year by year, and the original five cycles need to be divided into 2 + 3 cycles [18].

Therefore, the target rate of beds eventually owned by the elderly is assigned to each cycle. By 2020, the bed rate of the elderly per 1,000 registered residents in Nanjing is 45/1000, and it needs to reach 60/1000 by 2035. The specific targets for five cycles are 48/1000, 51/1000, 54/1000, 57/1000 and 60/1000 respectively. Starting from 2027, the proportion of smart community home care will increase, so the overall planning bed rate will also increase from the third cycle. For convenience of calculation, assuming that the annual increase rate of beds is 1/1000, the specific targets for the five cycles are 48/1000, 51/1000, 55/1000, 59/1000 and 63/1000 respectively. In this paper, the bed rate per thousand registered elderly is defined as the construction cost coefficient. The specific objectives are listed in Table 16, as follows.

**Table 16:** The achieve targets for specific beds in each cycle

Cycle	2021–2023	2024–2026	2027–2029	2030–2032	2033–2035
Original plan	48/1000	51/1000	54/1000	57/1000	60/1000
Existing Programs	48/1000	51/1000	55/1000	59/1000	63/1000

Combined with the previous institutional construction layout model, the objective function and bed ratio of each cycle are improved, and the following planning model of aged care institutions under the background of smart community is proposed. Since intelligent robots replace human services, the income of nursing homes will be reduced, and the proposed income will be reduced by half. Here are additional assumptions: Suppose that the efficiency of intelligent robot is twice that of human, so that the economic cost is halved, or the income coefficient is 0.5. Therefore, the objective function *F*_*1*_ is modified as follows:

9 $${F}_{1}=\mathrm{max }\sum_{i=1}^{2}\sum_{j=1}^{N}{b}_{ij}{x}_{ij}+0.5\left(\sum_{i=3}^{M}\sum_{j=1}^{N}{b}_{ij}{x}_{ij}\right)$$

At the same time, the model also needs to meet the following 10 constraints, and the mathematical model of the constraints is described as follows:

1. According to the specific bed realization goals in each cycle in Table 15, there are five constraints as follows:
  10 $$\begin{array}{c}\begin{array}{cc}\sum_{j=1}^Nd_{1j}+\sum_{j=1}^Nc_{1j}x_{1j}\geq0.048\ast{\mathrm P}_{2023},&\sum_{j=1}^Nd_{2j}+\sum_{j=1}^Nc_{2j}x_{2j}\geq0.051\ast{\mathrm P}_{2023}\end{array}\\\begin{array}{cc}\sum_{j=1}^Nd_{3j}+\sum_{j=1}^Nc_{3j}x_{3j}\geq0.055\ast{\mathrm P}_{2023}&\sum_{j=1}^Nd_{4j}+\sum_{j=1}^Nc_{4j}x_{4j}\geq0.059\ast{\mathrm P}_{2023}\end{array}\\\sum_{j=1}^Nd_{5j}+\sum_{j=1}^Nc_{5j}x_{5j}\geq0.063\ast{\mathrm P}_{2023}\end{array}$$
2. According to the elderly demand of Nanjing with low, lower, middle, upper and higher disposable income, there are five constraint conditions as follows:
  11 $$d_{ik}+\sum_{i=1}^Mc_{ij}x_{ik}\;\geq p_k\;\left(\sum_{i=1}^M\;\sum_{j=1}^N\;c_{ij}x_{ij}+\sum_j^Nd_{ij}\right),\;\left(i=1,2,3,4,5;\;k=1,2,3,4,5\right)$$

where *d*_*ij*_ is the total number of beds at *j*-level in the *i*-th cycle, and its calculation formula is:

12 $$d_{ij}=\sum_{i=1}^i\;d_{i-1,j}\;+\;\sum_{i=1}^i\;\sum_{j=1}^N\;c_{ij}x_{ij}$$

Taking Xuanwu District in Nanjing city as an example, based on the layout model of institutional construction, combined with the established planning model of aged care institutions, Matlab software was used to solve the problem. From 2027, intelligent robots will replace human services and the proportion of intelligent community home care will increase year by year, and the construction plan of intelligent pension trend in the next 15 years will be obtained, as shown in Table 17:

**Table 17:** The smart pension trend and number of aged care institutions in Xuanwu District

Year	A	AA	AAA	AAAA	AAAAA
2021–2023	15	5	1	1	1
2024–2026	31	8	1	1	2
2027–2029	40	12	1	2	2
2030–2032	54	27	1	4	3
2033–2035	73	21	2	4	4

According to the results, with the gradual increase of the elderly population in Nanjing, the proportion of intelligent home-based care will increase year by year since 2027, and the construction demand of aged care institutions will also increase. The number of construction at all levels rises gradually in each cycle, and the increase is more obvious after 2027. Therefore, it can be inferred that the planning model of aged care institutions construction is reasonable and more general.

## Discussion

By the end of 2019, the elderly population in Nanjing was about 880,000, accounting for 12.66% of the total population. The proportion of elderly population is relatively high, while showing the phenomenon of unbalanced regional development. Due to its long history, early development, high level of economic development and concentrated population distribution, the density of the elderly population in the old city is significantly higher than that in the outer urban areas and suburbs. According to statistics, the total land area of the old city only accounts for 3% of the city's total area, but it concentrates 36% of the city's elderly population. The high density of the elderly population poses a great challenge to the supporting services for the elderly. As of July 2019, there were only 245 nursing homes in Nanjing, most of which were small in scale and not fully equipped. There are less than 45 beds for every 1,000 registered elderly, which is difficult to meet the needs of the pension market and solve the problems of providing for, learning, enjoying and doing for the elderly.

Due to the prominent aging problem in Nanjing and the obvious differences among different regions, it is necessary to pay more attention to the problems of the elderly in the high-risk areas of aging and the inadequate development among different regions. According to the grade assessment results of aged care institutions released by Nanjing Civil Affairs Bureau in 2019, the construction proportion of 5A aged care institutions is relatively small. The reason may be that the land area of Xuanwu district, Qinhuai District and Gulou District is limited, while that of Liuhe District, Yuhuatai District, Lishui District and Gaochun District is limited by the economic level. All other districts have certain construction, and the situation of suburbs is obviously lower than that of the old city.

From the overall construction planning scheme given in this paper, the resources of A-grade aged care institutions in each district are relatively scarce. Therefore, in terms of future construction, more basic aged care institutions should be built to meet the pension needs of more people. The resources of A-grade aged care institutions in Jianye District, Yuhuatai District, Jiangning District and Lishui District are relatively scarce, which cannot meet the pension needs of low-income people. Therefore, it is recommended to increase investment in a-grade aged care institutions in these four areas. At the same time, it is suggested to pay more attention to the construction of aged care institutions in Xuanwu district, Qinhuai District, Gulou District, Liuhe District, Qixia District and Gaochun District. Since the proportion of elderly population in Xuanwu district, Gulou District and Qinhuai District is relatively high, it is suggested to strengthen the construction of aged care institutions to meet the needs of elderly population. Due to the relatively scarce resources of aged care institutions in Liuhe district and Gaochun District, the number of aged care institutions at all levels in these two regions should be accelerated in the case of a sharp rise in the demand for pension in the future. Qinhuai District has more available land area than the other two old urban areas, Xuanwu and Gulou, and the economic level of the old urban area is developed. Therefore, it is suggested to build three or four 5A-level aged care institutions every three years, while xuanwu and Gulou districts should build one 5A-level aged care institutions every three years. Gaochun District is located in the suburbs with wide usable area. It is suggested to build two 5A nursing institutions every three years on average. In addition, considering the large number of existing aged care institutions in Jianye District, Yuhuatai District, Pukou District, Jiangning District and Lishui District, more attention should be paid to the construction quality in the later construction. However, Xuanwu district, Qinhuai District, Gulou District, Liuhe District, Qixia District and Gaochun District have fewer aged care institutions, so more attention should be paid to the number of construction.

In recent years, the proportion of home care in smart communities is increasing. In the future, intelligent robots will replace human services, and the proportion will continue to rise. So the number of nursing home needs will still tend to rise. In order to achieve the balance between supply and demand, each district can implement the policy of assistance. The economically developed old city can invest in the construction of aged care institutions in the suburbs rich in land resources.

## Conclusions

At present, China is in the stage of demographic transition, and the population aging has developed rapidly in the past decade. How to establish a perfect social old-age service system has become a key issue of concern to the government and society. This paper combines the current situation of aged care institutions in each district of Nanjing, including the number of aged care institutions at all levels, the number of beds and so on. By constructing the multi-objective integer programming model, with the help of improved genetic algorithm and combined with multi-attribute decision analysis, the construction planning scheme of each district in Nanjing in the next 15 years and the construction scheme under the trend of intelligent endowment are given. Based on the research results, some suggestions on the construction planning of aged care institutions in Nanjing are put forward.

The construction of pension institutions has solved many pension problems of "three no old people", which can improve people's social happiness, promote social harmonious development and civilization progress. However, these indicators related to the construction of pension institutions are difficult to quantify. Subsequent studies can further transform such qualitative variables into numerical variables and add them to the social cost *F*_*2*_.

The solution of integer programming problem is limited to single objective optimization. As a classical algorithm for solving multi-objective optimization problems, the results of genetic algorithm are not integer. Therefore, this paper improved the genetic algorithm, combined with the TOPSIS method, the integer programming problem is applied to the genetic algorithm, the genetic algorithm integer programming problem. As for the layout and planning of aged care institutions, the construction scheme not only considers economic and environmental benefits, but also combines the current situation of aged care institutions in various districts of Nanjing. The model is effective and practical, and has realistic application value. At the same time, there is still room for improvement in the objective function of the model. More model solving algorithms can be further explored to give more possibilities for reference.

## Data Availability

The datasets used and analysed during the current study are available from the corresponding author on reasonable request.
