# Correction: Research on layout model and construction planning of aged care institutions for disabled elders in China: based on Nanjing city data

**DOI:** 10.1186/s12877-023-04047-1

**Published:** 2023-07-24

**Authors:** Guo Xiaojun, Shen Houxue, Wen Qinglan, Liu Sifeng, Yingjie Yang, Zhang Hui

**Affiliations:** 1 School of Science, Nantong University, 9 Seyuan Road, Nantong, 226019 China; 2 College of Economics and Management, Nanjing University of Aeronautics and Astronautics, Nanjing, 211106 China; 3 Institute of Artifcial Intelligence, De Montfort University, Leicester, LE1 9BH UK

Correction: BMC Geriatr 23, 237 (2023)

10.1186/s12877-023-03924-z

After publication of this article [1], the authors reported that, unfortunately, the author proof corrections had not been carried out.

The original article [1] has been corrected.
